# Antiplatelet effect of spironolactone via modulation of cPLA_2_-mediated thromboxane A_2_ generation in murine platelets

**DOI:** 10.4142/jvs.26033

**Published:** 2026-08-20

**Authors:** Sanggu Kim, Preeti Kumari Chaudhary, Seongmo Yang, Soochong Kim

**Affiliations:** 1 Laboratory of Veterinary Pathology and Platelet Signaling, College of Veterinary Medicine, Chungbuk National University, Cheongju 28644, Korea.; 2 Molecular Cardiology Research Institute, Tufts University School of Medicine, Boston, MA 02111, USA.; 3 Cancer Research Center, Chungbuk National University, Cheongju 28644, Korea.

**Keywords:** Mineralocorticoid receptor antagonists, signal transduction, arachidonic acid, phospholipase A_2_, thrombosis

## Abstract

**Importance:**

Spironolactone, a steroidal aldosterone antagonist, is widely used as a diuretic and antihypertensive agent. Steroids have been known to promote thrombosis by directly increasing platelet count and activation and indirectly by increasing oxidative stress and suppressing nitric oxide synthesis. However, the effects of spironolactone on platelet activation remain unclear.

**Objective:**

To investigate the effects of spironolactone on platelet function and underlying signaling mechanisms.

**Methods:**

Platelet aggregation, dense granule secretion, thromboxane A_2_ (TxA_2_) generation, and protein phosphorylation were evaluated in platelets treated with spironolactone.

**Results:**

Spironolactone significantly inhibited the secondary wave of platelet aggregation and dense granule secretion induced by 2-methylthioadenosine diphosphate (2-MeSADP). It is known that 2-MeSADP-induced secretion and the resultant secondary wave of aggregation depend on TxA_2_-mediated positive feedback, suggesting a role for spironolactone in regulating TxA_2_ generation in platelets. Additionally, spironolactone did not affect 2-MeSADP-induced platelet aggregation in aspirin-treated platelets in which TxA_2_ generation was blocked. Furthermore, platelet aggregation and secretion induced by low concentrations of thrombin, which are also affected by TxA_2_-mediated positive feedback, were partially inhibited by spironolactone. Notably, spironolactone significantly suppressed TxA_2_ generation induced by both 2-MeSADP and thrombin. Finally, spironolactone markedly inhibited the 2-MeSADP- and thrombin-induced phosphorylation of cytosolic phospholipase A_2_ (cPLA_2_), extracellular signal-regulated kinase, and protein kinase B in non-aspirin-treated platelets, whereas only cPLA_2_ phosphorylation was significantly inhibited in aspirin-treated platelets.

**Conclusions and Relevance:**

Spironolactone inhibits platelet activation by suppressing TxA_2_ generation, primarily by modulating cPLA_2_ phosphorylation, providing mechanistic insight into its effects on platelet signaling *in vitro*.

## INTRODUCTION

Platelets play a central role in hemostasis and coagulation following vascular injury and bleeding. Exposure to subendothelial collagen and von Willebrand factor at the vascular damage site promotes platelet adhesion and activation through their interactions with the platelet receptors glycoproteins VI and Ib [1]. Activated platelets subsequently release various granule-derived mediators, including thromboxane A_2_ (TxA_2_), adenosine diphosphate (ADP), and serotonin, which recruit additional platelets and promote platelet aggregation, thereby forming a primary hemostatic plug [2]. Platelets provide a catalytic surface for the final steps of the intrinsic and extrinsic coagulation pathways, thereby facilitating thrombin generation and fibrinogen conversion to fibrin [3]. The resulting fibrin mesh stabilizes the platelet plug, forming a durable hemostatic thrombus.

However, dysregulation of platelet function, whether excessive or impaired, markedly increases the risk of thrombotic and hemorrhagic disorders. Thrombotic diseases affect approximately 0.2% of the global population annually [4]. Deep vein thrombosis is associated with a 90-day mortality rate of approximately 13.75%, whereas myocardial infarction and stroke have a 30-day mortality rate of approximately 10%, indicating substantial clinical lethality [5,6]. Although bleeding disorders are relatively rare, with approximately 400,000 registered patients worldwide, they are frequently characterized by the absence of prodromal symptoms, which can result in sudden physiological collapse and unexpected death, leading to a high 30-day mortality rate [7]. Although antiplatelet and anticoagulant therapies have been used to prevent and treat these conditions, morbidity and mortality remain high. Therefore, a comprehensive investigation of platelet function and its underlying signaling mechanisms is vital in improving the prevention and management of thrombotic and hemorrhagic diseases.

Spironolactone, a steroidal aldosterone antagonist, is a competitive inhibitor of the mineralocorticoid receptor (MR) and has high binding affinity for MR and androgen receptors (ARs). It is a clinically indispensable therapeutic agent prescribed to millions of patients worldwide annually. By inhibiting MR-mediated signaling, spironolactone downregulates Na^+^/K^+^ channel expression, thereby exerting potent diuretic and antihypertensive effects [8]. Consequently, spironolactone is widely used for the clinical management of hypertension, heart failure, ascites, and chronic kidney disease [9] and is frequently prescribed to attenuate excessive aldosterone activity in patients with aldosteronism arising from adrenal hyperplasia or neoplasms [10].

However, excessive spironolactone administration is associated with adverse hemodynamic and hematological effects, including hypotension secondary to diuretic-induced hemoconcentration and hemostatic abnormalities, such as thrombocytopenia and bleeding [11,12]. Despite these clinically relevant adverse events and the extensive use of spironolactone in diverse patient populations, its effects on platelet function and the underlying platelet signaling pathways remain unclear.

This study demonstrated the effect of spironolactone on platelet function and its underlying mechanism using washed murine platelets. Spironolactone inhibits 2-methylthioadenosine diphosphate (2-MeSADP)- and low-concentrations of thrombin-induced platelet secretion and the resultant secondary wave of aggregation. In aspirinated platelets, spironolactone had no effect on platelet aggregation and secretion induced by 2-MeSADP and low concentrations of thrombin, suggesting that spironolactone exerts its antiplatelet effect by regulating TxA_2_ generation. Furthermore, 2-MeSADP- and thrombin-induced cytosolic phospholipase A_2_ (cPLA_2_), extracellular signal-regulated kinase (ERK), and protein kinase B (AKT) phosphorylation were inhibited by spironolactone; however, only cPLA_2_ phosphorylation was inhibited in aspirinated platelets. Thus, spironolactone affects platelet function by inhibiting TxA_2_ generation primarily through the modulation of the signaling pathway regulating cPLA_2_ phosphorylation.

## METHODS

### Materials

The 2-MeSADP, thrombin, apyrase (type V), prostaglandin E_1_ (PGE_1_), and sodium citrate were purchased from Sigma-Aldrich (USA). The luciferin-luciferase reagent was purchased from ChronoLog (USA). The thromboxane B_2_ (TxB_2_) enzyme-linked immunosorbent assay (ELISA) kit was purchased from Enzo Life Sciences (UK). Anti-phospho-AKT (Ser473), anti-AKT, anti-phospho-ERK (Thr202/Tyr204), anti-ERK, anti-phospho-cPLA_2_ (Ser505), anti-cPLA_2_, anti-rabbit IgG, and horseradish peroxidase-linked antibodies were purchased from Cell Signaling Technology (USA). SuperSignal West Pico PLUS Chemiluminescent Substrate was purchased from Thermo Scientific (USA). All the other reagents were of reagent grade.

### Washed platelet preparation

Whole blood was collected from 8-week-old male C57BL/6 mice by cardiac puncture using 3.8% sodium citrate as an anticoagulant. To minimize biological variability associated with hormonal fluctuations known to influence platelet function, only male mice were used in this study. All animals were deeply anesthetized with isoflurane prior to blood collection, and all procedures were performed in accordance with the American Veterinary Medical Association Guidelines for the Euthanasia of Animals. The collected blood was centrifuged at 100 × g for 10 min to obtain platelet-rich plasma (PRP). To prevent platelet activation, PRP was incubated with PGE_1_ (1 µM) at room temperature for 10 min. In the aspirin-treated group, PRP was incubated with aspirin (1 mM) at 37°C for 30 min. The plasma fraction was centrifuged at 400 × g for 10 min to pellet the platelets. The platelet pellet was resuspended in Tyrode’s buffer (pH 7.4) containing apyrase (0.05 units/mL), and platelet counts were determined. No predefined inclusion or exclusion criteria were applied, and all samples were included in the analysis. Randomization and blinding were not applicable because experiments were conducted with isolated platelets under controlled *in vitro* conditions and used objective, instrument-based measurements.

### Platelet aggregation and secretion

Agonist-induced platelet aggregation and dense granule secretion were assessed using a Lumi-aggregometer (ChronoLog) at 37°C with constant stirring at 900 rpm [13]. Briefly, washed platelets were preincubated with either 0.5 µL of vehicle (ethanol, control) or 0.5 µL of ethanol containing spironolactone (final concentration; 50 µM) for 5 min before agonist stimulation based on previous *in vitro* experiments [14,15,16,17]. Washed platelets were adjusted to a final concentration of 2 × 10^8^ platelets/mL, and 250 µL aliquots were supplemented with luciferin–luciferase before stimulation with platelet agonists. Platelet aggregation and dense granule secretion were simultaneously monitored by measuring the changes in light transmission and luminescence.

### TxA_2_ generation

After 3.5 min of platelet activation, reactions were terminated by rapid freezing in liquid nitrogen. Samples were stored at −80°C until TxB_2_ analysis. The samples were gently thawed and centrifuged at 3,000 × g for 10 min. The supernatants were diluted 1:20 with the assay buffer, and TxB_2_ levels were measured in duplicate using a TxB_2_ ELISA kit according to the manufacturer’s instructions.

### Western blotting

Washed platelets (2 × 10^8^ platelets/mL) were stimulated with agonists for 2 min at 37°C, and reactions were terminated by adding 6.6 N perchloric acid. Samples were centrifuged at 3,000 × g for 10 min, and the pellets were washed twice with distilled water and resuspended in 2× Laemmli sample buffer (Bio-Rad Laboratories, USA). The samples were denatured at 100°C for 10 min and stored at −20°C until Western blot analysis.

Proteins were separated by sodium dodecyl sulfate-polyacrylamide gel electrophoresis on 10% polyacrylamide gels and transferred to nitrocellulose membranes. The membranes were blocked and incubated overnight at 4°C, with primary antibodies against phospho-AKT (Ser473), total AKT, phospho-ERK (Thr202/Tyr204), total ERK, phospho-cPLA_2_ (Ser505), and total cPLA_2_. The membranes were then incubated with the appropriate secondary antibodies for 1 h at room temperature. After washing, the immunoreactive bands were visualized using a chemiluminescent substrate and captured using an iBright 1500 Imaging System (Thermo Fisher Scientific, USA).

### Statistical analysis

All statistical analyses were conducted using the Prism software (version 9.1; GraphPad Software, USA). Data are presented as mean ± standard error. Statistical significance was assessed using the paired Student’s *t*-test or one-way analysis of variance (ANOVA). Global ANOVA *p* values were reported before *post-hoc* Bonferroni correction. Normality was evaluated using the Shapiro–Wilk test, and all datasets were confirmed to follow a normal distribution (*p* > 0.05). Exact *p* values are reported, and 95% confidence intervals were calculated for key comparisons. A two-sided α level of 0.05 was considered statistically significant.

## RESULTS

### Spironolactone inhibits 2-MeSADP-induced platelet aggregation and secretion only in non-aspirin-treated platelets

To investigate the role of spironolactone in platelet function, platelets were stimulated with 2-MeSADP, and platelet aggregation and dense granule secretion were assessed. Spironolactone at 50 µM markedly inhibited the 2-MeSADP-induced platelet aggregation and dense granule secretion (Fig. 1). In contrast, when platelets were pretreated with aspirin, the cyclooxygenase (COX) inhibitor, spironolactone had no additional effect on platelet aggregation or dense granule secretion.

**Fig. 1:**
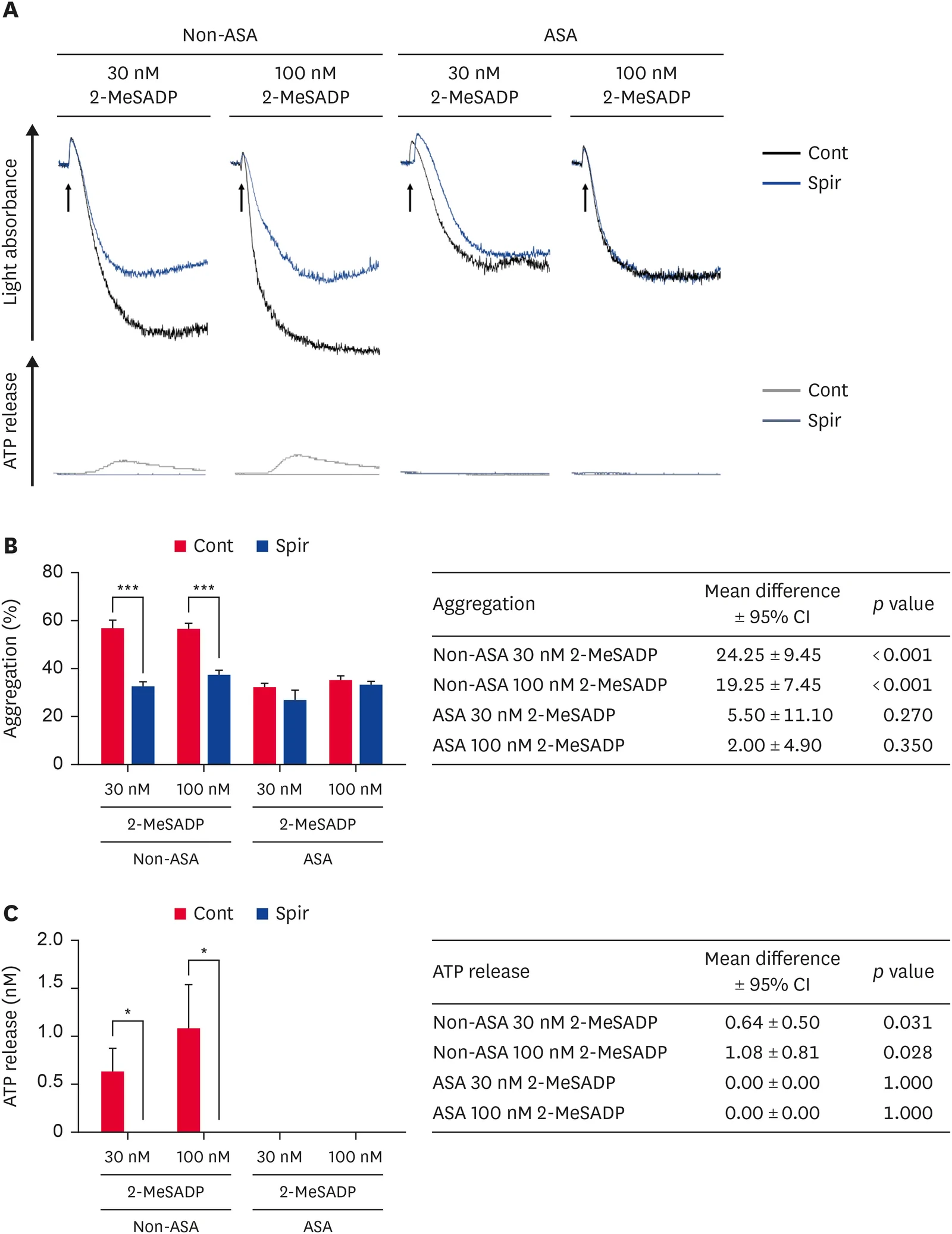
The effect of spironolactone on 2-MeSADP-induced platelet aggregation and secretion in non-aspirin-treated and aspirin-treated platelets. (A) Non-aspirin-treated and aspirin-treated washed platelets were stimulated with 30 or 100 nM 2-MeSADP with or without 50 µM spironolactone, and platelet aggregation and secretion were measured (n = 5). The extent of (B) platelet aggregation and (C) dense granule secretion were quantified from panel A. Data are shown as mean ± standard error. 2-MeSADP, 2-methylthioadenosine diphosphate; ASA, acetylsalicylic acid; CI, confidence interval. ^*^p < 0.05, ^***^p < 0.001.

### Spironolactone inhibits low concentrations of thrombin-induced platelet aggregation and secretion only in non-aspirin-treated platelets

To assess the role of spironolactone in protease-activated receptor (PAR)-mediated platelet function, platelets were stimulated with thrombin. Similar to the results observed with 2-MeSADP, spironolactone markedly inhibited platelet aggregation and dense granule secretion induced by low concentrations of thrombin (Fig. 2). In contrast, stimulation with high concentrations of thrombin resulted in only a modestly reduced dense granule secretion without affecting platelet aggregation. Furthermore, when platelets were pretreated with aspirin, spironolactone had no additional effect on thrombin-induced platelet aggregation or dense granule secretion.

**Fig. 2:**
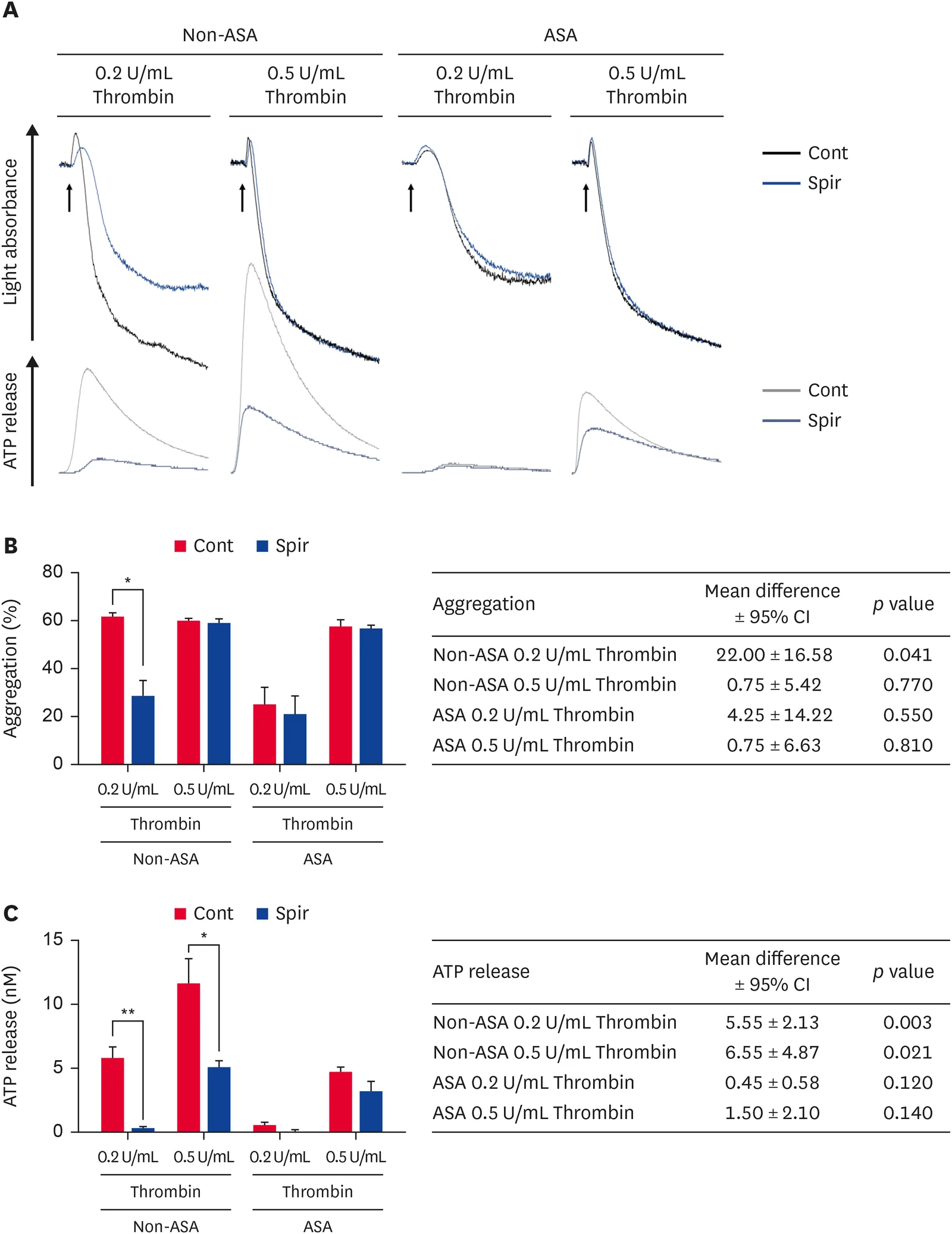
The effect of spironolactone on thrombin-induced platelet aggregation and secretion in non-aspirin-treated and aspirin-treated platelets. (A) Non-aspirin-treated and aspirin-treated washed platelets were stimulated with 0.2 and 0.5 U/mL thrombin with or without 50 µM spironolactone, and platelet aggregation and secretion were measured (n = 5). The extent of (B) platelet aggregation and (C) dense granule secretion were quantified from panel A. Data are shown as mean ± standard error. ASA, acetylsalicylic acid; CI, confidence interval. ^*^p < 0.05, ^**^p < 0.01.

### Spironolactone inhibits 2-MeSADP- and thrombin-induced platelet TxA_2_ generation

To further evaluate the effect of spironolactone on TxA_2_ generation, platelets were stimulated with 2-MeSADP or thrombin, and TxA_2_ generation was quantified. Spironolactone significantly reduced the TxA_2_ generation induced by both agonists, demonstrating its critical role in regulating TxA_2_ production (Fig. 3).

**Fig. 3:**
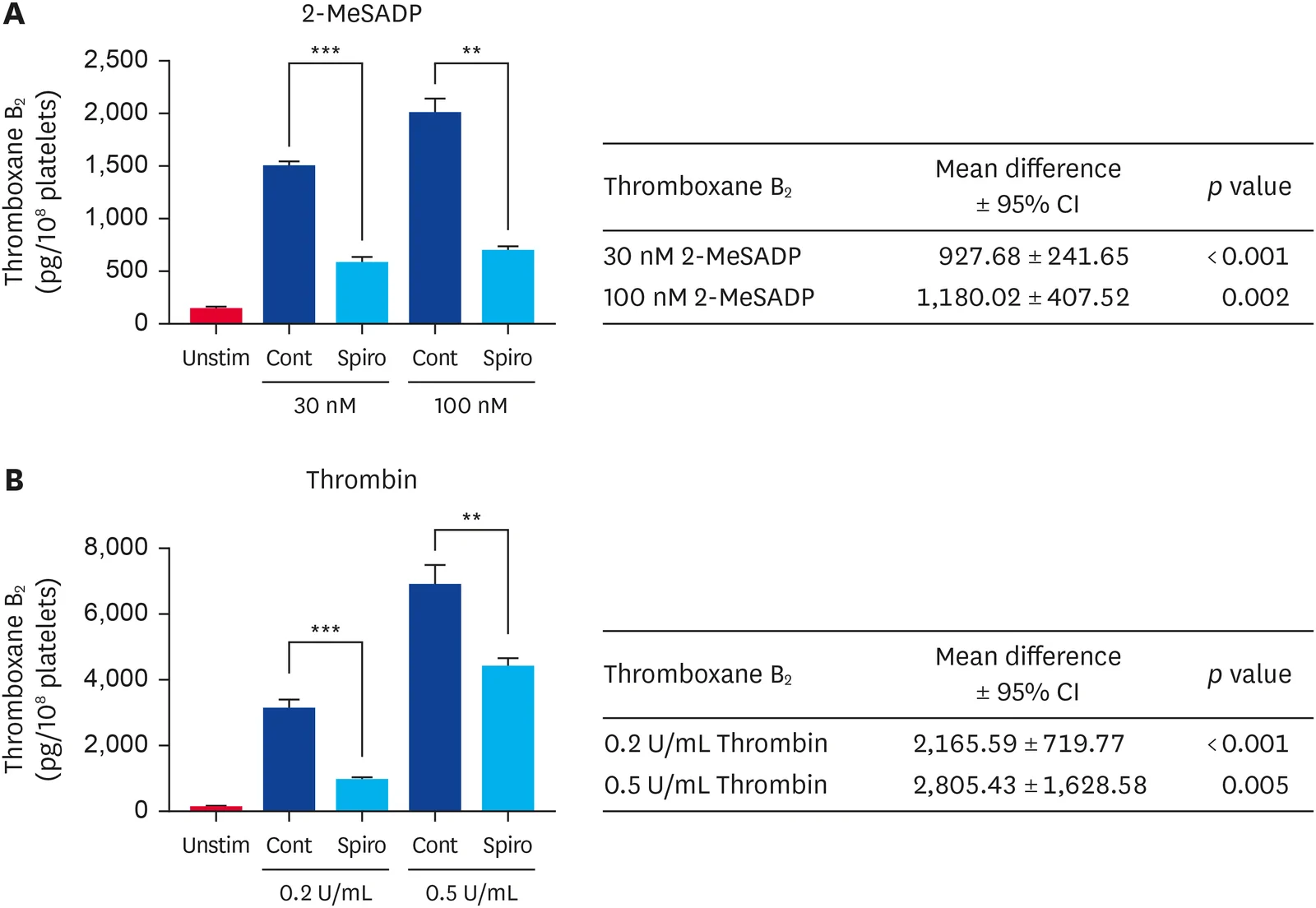
The effect of spironolactone on 2-MeSADP- and thrombin-induced platelet TxA_2_ generation. Washed platelets were stimulated with (A) 2-MeSADP and (B) thrombin for 3.5 min and TxB_2_ generation was measured (n = 6). Data are shown as mean ± standard error. 2-MeSADP, 2-methylthioadenosine diphosphate; TxA_2_, thromboxane A_2_; CI, confidence interval. ^**^p < 0.01, ^***^p < 0.001.

### Spironolactone inhibits 2-MeSADP-induced platelet cPLA_2_, ERK, and AKT phosphorylation

To determine the molecular mechanisms underlying the regulation of TxA_2_ generation by spironolactone, platelets were stimulated with 2-MeSADP in the presence or absence of aspirin, and the phosphorylation of cPLA_2_, ERK, and AKT was assessed. The 2-MeSADP-induced phosphorylation of cPLA_2_, ERK, and AKT was markedly inhibited by spironolactone in non-aspirin-treated platelets (Fig. 4). In contrast, in aspirin-treated platelets, ERK and AKT phosphorylation was attenuated and showed no additional reduction upon spironolactone treatment, whereas cPLA_2_ phosphorylation was not inhibited by aspirin but was markedly reduced by spironolactone.

**Fig. 4:**
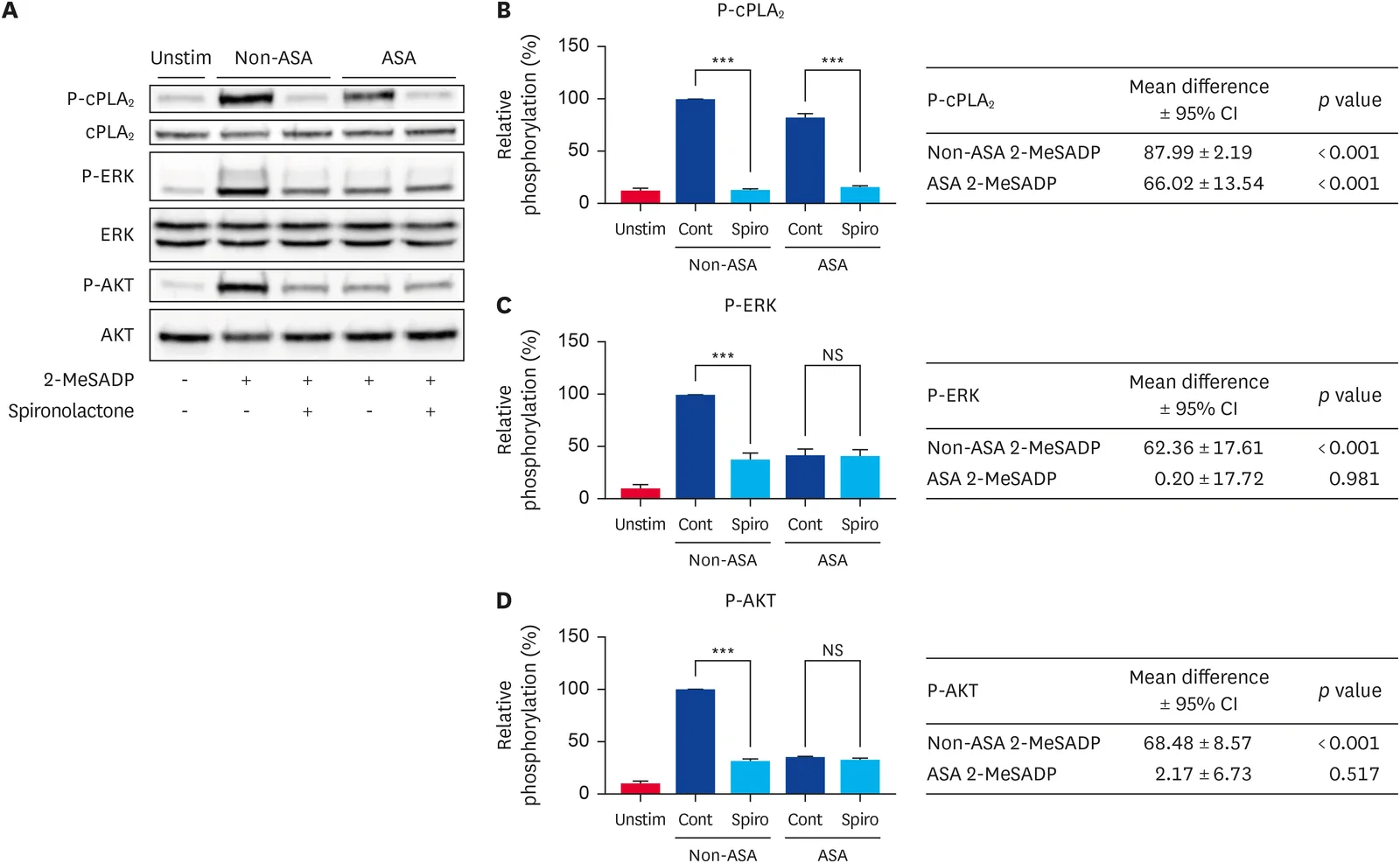
The effect of spironolactone on 2-MeSADP-induced platelet cPLA_2_, ERK, and AKT phosphorylation. (A) Washed platelets were stimulated with 2-MeSADP for 2 min, and phosphorylation of cPLA_2_, ERK, and AKT was measured (n = 3). The extent of relative phosphorylation of (B) cPLA_2_, (C) ERK, and (D) AKT was quantified from panel A. Data are shown as mean ± standard error. 2-MeSADP, 2-methylthioadenosine diphosphate; cPLA_2_, cytosolic phospholipase A_2_; ERK, extracellular signal-regulated kinase; AKT, protein kinase B; P-, phosphorylation of; ASA, acetylsalicylic acid; CI, confidence interval. ^***^p < 0.001, ^NS^*p* > 0.05. NS, not significant.

### Spironolactone inhibits thrombin-induced platelet cPLA_2_, ERK, and AKT phosphorylation

To evaluate the effects of spironolactone on other agonist-induced platelet molecular mechanisms, the platelets were stimulated with thrombin in the presence of spironolactone. Similar to the findings observed with 2-MeSADP, thrombin-induced phosphorylation of cPLA_2_, ERK, and AKT was significantly inhibited by spironolactone in non-aspirin-treated platelets (Fig. 5). In aspirin-treated platelets, ERK and AKT phosphorylation was inhibited by aspirin and was not further reduced by spironolactone. In contrast, cPLA_2_ phosphorylation was not inhibited by aspirin but was significantly suppressed by spironolactone.

**Fig. 5:**
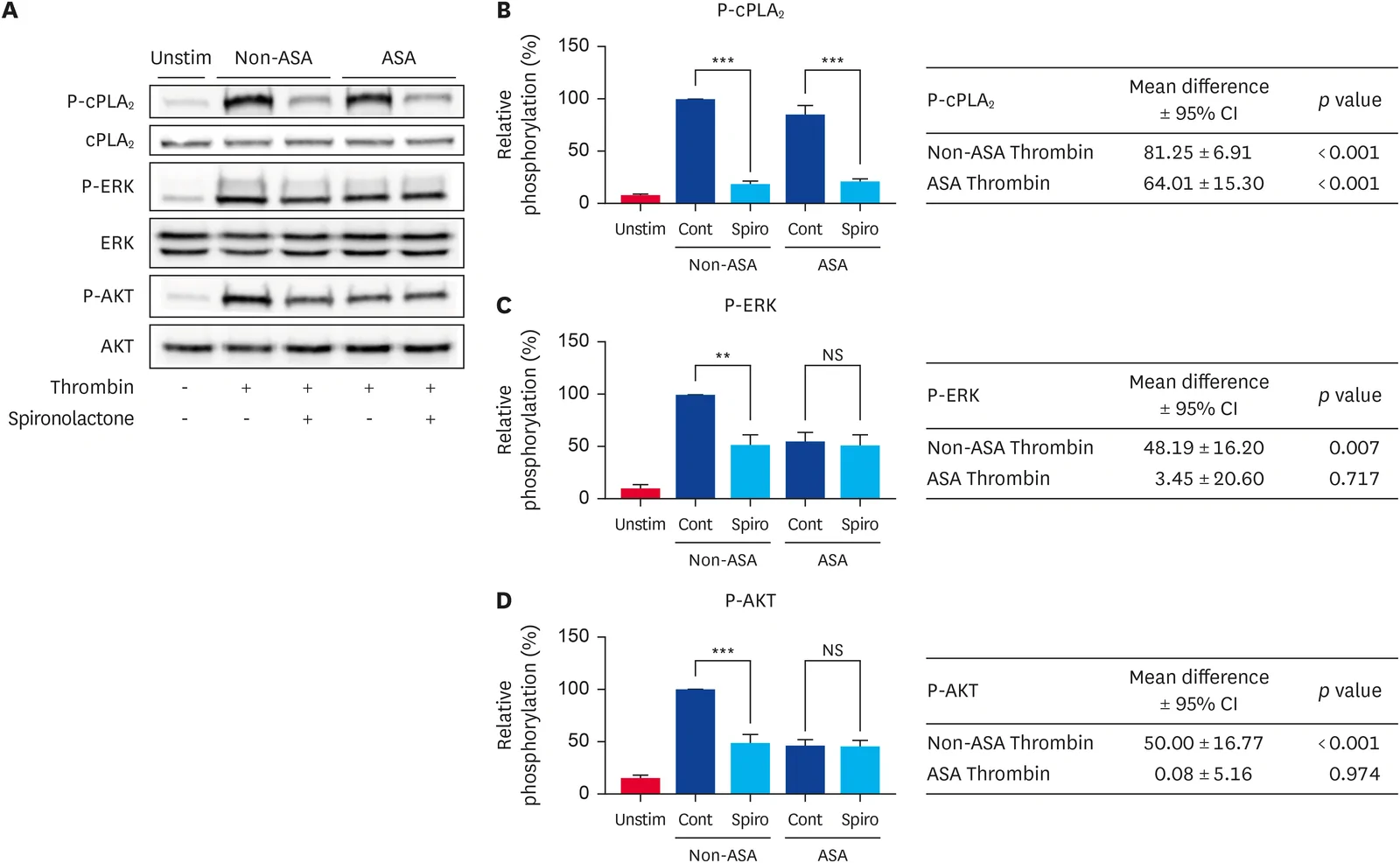
The effect of spironolactone on thrombin-induced platelet cPLA_2_, ERK, and AKT phosphorylation. (A) Washed platelets were stimulated with thrombin for 2 min, and phosphorylation of cPLA_2_, ERK, and AKT was measured (n = 3). The extent of relative phosphorylation of (B) cPLA_2_, (C) ERK, and (D) AKT was quantified from panel A. Data are shown as mean ± standard error. cPLA_2_, cytosolic phospholipase A_2_; ERK, extracellular signal-regulated kinase; AKT, protein kinase B; P-, phosphorylation of; ASA, acetylsalicylic acid; CI, confidence interval, NS, not significant. ^**^p < 0.01, ^***^p < 0.001, and ^NS^*p* > 0.05.

## DISCUSSION

Spironolactone, a widely prescribed MR antagonist, has long been used to treat heart failure, hypertension, primary aldosteronism, and other cardiovascular disorders [9]. Spironolactone is a valuable therapeutic agent in clinical practice because of its potassium-sparing diuretic and cardioprotective properties. However, its use has been associated with several adverse effects, including hypotension, thrombocytopenia, and bleeding [11,12]. Despite extensive clinical use, its direct effects on platelet function and underlying intracellular signaling mechanisms remain unclear. Considering the pivotal role of platelets in thrombosis and cardiovascular disease, a more detailed understanding of platelet activation modulation by spironolactone is required. This study aimed to investigate the effects of spironolactone on platelet function and delineate its underlying signaling pathways.

In this study, spironolactone markedly suppressed platelet function. Spironolactone significantly inhibited 2-MeSADP-induced platelet aggregation and secretion. ADP-induced platelet aggregation is known to consist of secretion-independent primary aggregation and secretion-dependent secondary aggregation driven by generated TxA_2_ [18]. Notably, spironolactone completely abolished platelet secretion while selectively suppressing secondary aggregation. This pattern strongly suggests that spironolactone inhibits platelet activation by suppressing the TxA_2_-dependent feedback effect rather than by directly inhibiting the initial ADP signaling. This hypothesis was supported by experiments using aspirin, a COX inhibitor that blocks TxA_2_ generation. In aspirin-treated platelets, spironolactone exerted no additional effects on aggregation or secretion, indicating that its inhibitory effect targeted TxA_2_ generation.

To determine the effects of spironolactone on other agonist-induced platelet activation, we examined platelet responses to thrombin, another G protein-coupled receptor (GPCR) agonist that activates platelets through PARs. Spironolactone inhibited both platelet aggregation and secretion induced by low concentrations of thrombin, which was consistent with its effects on ADP-induced platelet activation. In contrast, when platelets were stimulated with high concentrations of thrombin, spironolactone slightly decreased secretion without affecting platelet aggregation. This differential effect is consistent with previous reports demonstrating that high-concentration thrombin-induced platelet aggregation is less dependent on TxA_2_-mediated feedback than ADP-induced platelet aggregation [19]. Importantly, spironolactone did not show any additional inhibitory effect on thrombin-induced platelet aggregation and secretion in aspirin-treated platelets, which is consistent with the observations obtained under 2-MeSADP stimulation. Moreover, direct measurement of TxA_2_ confirmed that spironolactone significantly reduced TxA_2_ generation. Collectively, these findings provide strong evidence that spironolactone suppresses GPCR-mediated platelet activation, primarily by inhibiting TxA_2_ generation.

TxA_2_ generation is a central amplification mechanism in platelet activation and is tightly regulated by intracellular signaling cascades. Upon platelet stimulation, cPLA_2_ phosphorylation promotes the release of arachidonic acid, which is converted to TxA_2_ by COX-1 [20]. In this study, spironolactone significantly inhibited phosphorylation of cPLA_2_, ERK, and AKT in platelets stimulated with either 2-MeSADP or thrombin. To further clarify the direct effector of spironolactone involved in the TxA_2_ signaling cascade, we examined these pathways in aspirin-treated platelets. Agonist-induced ERK and AKT phosphorylation was markedly suppressed in aspirin-treated platelets, and spironolactone treatment showed no additional reduction, indicating that ERK and AKT phosphorylation are dependent on TxA_2_-mediated positive feedback effect. However, agonist-induced cPLA_2_ phosphorylation was maintained in aspirin-treated platelets but was significantly inhibited by spironolactone treatment, demonstrating that spironolactone directly regulates cPLA_2_ activation, which is the upstream regulator of TxA_2_ generation. Together, these findings suggest that spironolactone regulates TxA_2_ generation primarily through the direct inhibition of cPLA_2_ phosphorylation, while the observed effects on ERK and AKT are likely secondary to reduced TxA_2_-dependent feedback signaling.

Spironolactone primarily exerts its pharmacological effects by antagonizing the MR and, to a lesser extent, the AR. Both MR and AR have been reported to be expressed in platelets [21,22]. Because platelets are anucleate cells, MR in platelets cannot function as a conventional nuclear transcription factor; however, it participates in non-genomic signaling pathways localized to the cytoplasm or plasma membrane [23]. Such non-genomic signaling does not involve nuclear transcription or translation; therefore, it can elicit rapid cellular responses within minutes of stimulation [24]. In various nucleated cell types, MR activation has been shown to rapidly stimulate the Src-mitogen-activated protein kinases (MAPK) and phosphatidylinositol 3-kinase (PI3K)-AKT signaling pathways, whereas pharmacological inhibition of MR attenuates Src-MAPK and PI3K-AKT activation [25,26]. The concentration of spironolactone used in this study (50 µM) is sufficient to achieve complete MR antagonism in most cell types [15,16,17,24]. However, under physiological conditions, platelet MR may operate in a tonic state of activation [27]. Therefore, although MR inhibition in platelets can modulate Src-MAPK or PI3K-AKT signaling, MR inhibition alone would be insufficient to suppress Src-MAPK or PI3K-AKT pathways via agonist-induced platelet activation in this study. However, phosphorylation of cPLA_2_ was markedly reduced by spironolactone treatment. This observation raises the possibility that spironolactone may suppress cPLA_2_ activation via mechanisms independent of non-genomic MR-mediated signaling, either by directly inhibiting cPLA_2_ phosphorylation or by engaging alternative regulatory pathways upstream of cPLA_2_ phosphorylation.

One important consideration in the present study is that the concentration of spironolactone used (50 µM) is substantially higher than the reported therapeutic plasma concentrations in humans. In humans, spironolactone is typically administered at doses ranging from 50 to 400 mg for the treatment of hyperaldosteronism and as a diuretic. Reported peak plasma concentrations of spironolactone following oral administration are 48.34 ng/mL (116 nM) after a 100 mg dose and 185 ng/mL (441.1 nM) after a 200 mg dose [15,28], which remain more than 100-fold lower than the 50 µM concentration used in the present study. However, similar micromolar ranges (approximately 10–100 µM) have been widely used in previous *in vitro* studies to evaluate the direct cellular effects of spironolactone, including its anti-inflammatory, antioxidant, and antiproliferative actions across various cell types [15,16,17]. In our study, spironolactone produced clear inhibitory effects at 50 µM. These findings suggest that relatively higher concentrations may be required to directly modulate platelet function under isolated *in vitro* conditions. Therefore, the present results should be interpreted as mechanistic insights into how spironolactone can regulate platelet signaling pathways, rather than as direct evidence of equivalent effects at clinically relevant concentrations.

Overall, our study demonstrates that spironolactone acts as a potent inhibitor of platelet function by inhibiting TxA_2_ generation primarily through the modulation of cPLA_2_ phosphorylation. These findings provide mechanistic insight into the modulation of platelet signaling by spironolactone under *in vitro* conditions. Further studies are needed to determine whether similar effects occur at clinically relevant concentrations or *in vivo*.
